# Effect of a novel endoscope cleaning brush on duodenoscope contamination

**DOI:** 10.1055/a-2193-4481

**Published:** 2023-12-05

**Authors:** Koen van der Ploeg, Cynthia P. Haanappel, Anne F. Voor in ’t holt, Woutrinus de Groot, Adriana J.C. Bulkmans, Nicole S. Erler, Bibi C.G.C. Mason-Slingerland, Margreet C. Vos, Marco J. Bruno, Juliëtte A. Severin

**Affiliations:** 1 6993 Department of Gastroenterology and Hepatology, Erasmus MC University Medical Center Rotterdam, Rotterdam, Netherlands; 2 6993 Department of Medical Microbiology and Infectious Diseases, Erasmus MC University Medical Center Rotterdam, Rotterdam, Netherlands; 3 6993 Quality Assurance and Regulatory Affairs office Medical Technology, Erasmus MC University Medical Center Rotterdam, Rotterdam, Netherlands; 4 6993 Department of Biostatistics, Erasmus MC University Medical Center Rotterdam, Rotterdam, Netherlands; 5 6993 Department of Epidemiology, Erasmus MC University Medical Center Rotterdam, Rotterdam, Netherlands

## Abstract

**Background** Current duodenoscope reprocessing protocols are insufficient to prevent contamination and require adaptations to prevent endoscopy-associated infections (EAIs). This study aimed to investigate the effect of a new endoscope cleaning brush on the contamination rate of ready-to-use duodenoscopes.

**Methods** This retrospective before-and-after intervention study collected duodenoscope surveillance culture results from March 2018 to June 2022. Contamination was defined as ≥1 colony-forming unit of a microorganism of gut or oral origin (MGO). In December 2020, an endoscope cleaning brush with a sweeper design was introduced as an intervention in the manual cleaning of duodenoscopes. A logistic mixed-effects model was used to study the effects of this intervention.

**Results** Data were collected from 176 culture sets before the new brush’s introduction and 81 culture sets afterwards. Pre-introduction, culture sets positive with an MGO comprised 45.5% (95%CI 38.3%–52.8%; 80/176), decreasing to 17.3% (95%CI 10.6%–26.9%; 14/81) after implementation of the new brush. Compared with the former brush, duodenoscopes cleaned with the new brush had lower odds of contamination with MGOs (adjusted odds ratio 0.25, 95%CI 0.11–0.58; *P* =0.001)

**Conclusions** Use of the new brush in manual cleaning reduced contamination with MGOs and is expected to prevent EAIs. These findings should be confirmed in future prospective randomized studies.

## Abstract (de)

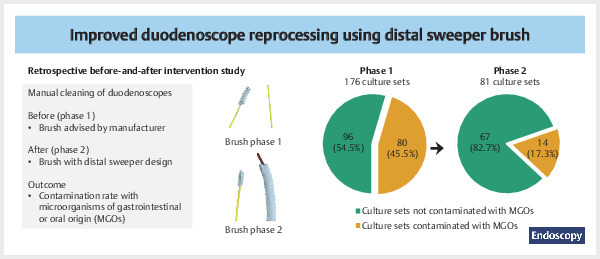

## Introduction

Infection is a potential (severe) complication of endoscopic retrograde cholangiopancreatography (ERCP), occurring in 1.4%–7.7% of patients, with a mortality rate of 7.8% 1 2 . Infectious complications post-ERCP can result from the translocation of endogenous intestinal flora during the procedure or the introduction of exogenous microorganisms via contaminated equipment. Contaminated duodenoscopes have caused multiple nosocomial outbreaks, mainly involving multidrug-resistant organisms, resulting in cases of illness and death 3 . Studies on duodenoscope contamination rates show significant variation. A recent meta-analysis reported a contamination rate of 21.5% (95%CI 15.4%–27.6%) in nonoutbreak-initiated studies 4 .

A major factor responsible for duodenoscope contamination is biofilm formation. Risk factors for biofilm formation include reprocessing lapses, delays before reprocessing, endoscope damage, and insufficient drying 5 . Biofilms can reduce the efficacy of high level disinfection (HLD) and may cause false-negative culture results 5 6 7 . Once a biofilm has formed in the endoscope channels, it is difficult to remove and may require channel replacement 8 .

Manual cleaning of duodenoscopes is considered a critical step in achieving adequate reprocessing and involves flushing and brushing of endoscope channels 9 . Currently, the duodenoscope channel cleaning brushes advised by the duodenoscope manufacturers consist of a wire with a single cleaning brush. However, an in vitro study demonstrated that the Endoss “Push and Pull” brush (EPP; JPP50, Endoss BV), a cleaning brush with a sweeper design, might be more efficient in cleaning duodenoscope channels 10 . In this study, we aimed to evaluate the effect of EPP introduction on the contamination rate of Pentax ED34-i10T2 duodenoscopes.

## Methods

### Setting

This retrospective before-and-after intervention study was performed in a large tertiary care center, the Erasmus MC University Medical Center (Erasmus MC), Rotterdam, The Netherlands, where approximately 750 ERCP procedures are performed on adult patients annually. We included culture sets collected from eight Pentax ED34-i10T2 duodenoscopes (with disposable caps) from March 2018 until June 2022. Reprocessing was performed by dedicated reprocessing staff according to the manufacturer’s instructions.

### Intervention

**Fig. 1:**
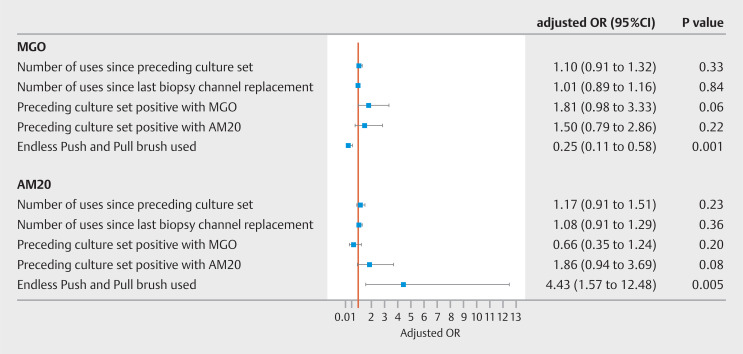
Forrest plot with results of mixed-model analysis of duodenoscope culture sets by contamination definition. OR, odds ratio; MGO, microorganism of gut or oral origin; AM20, any microbial growth with ≥20 colony forming units/20 mL, including waterborne or skin-type microorganisms.

On December 15, 2020, the EPP was introduced for manual cleaning of the Pentax ED34-i10T2 duodenoscopes and replaced the Pentax single-use brush (CS5522A) ( **Fig. 1s** , see online-only supplementary material).

### Sampling

The duodenoscope culture sets consisted of five sample sites. First, the distal tip of the duodenoscope was swabbed using a Copan Liquid Amies Elution Swab (eSwab; Copan). Then 20 mL of sterile saline (0.9%) was flushed through each of the suction channel, biopsy channel, and air water channel and collected separately in sterile containers. Subsequently, a single-use endoscope cleaning brush (Pentax CS5522A) was pulled through the suction and biopsy channels. The distal tip of the brush was cut using disinfected pliers and placed in an eSwab container.

Starting in April 2021, sterile water was used as the flushing fluid instead of saline. Routine surveillance cultures were taken approximately monthly. Data on the exact timing of sampling and errors in the sampling process were not available.

### Microbiological methods and interpretation

The eSwab containers were vortexed and poured over a sheep blood agar plate (Becton Dickinson). The flushing fluid was filtered through a 0.22-µm filter (Milliflex Plus Test System), after which the filter was placed on Reasoners2A agar (Becton Dickinson). Plates were incubated for 3 days at 35°C. All morphologically distinct microorganisms were identified and colony-forming units (CFUs) were counted. Identification was performed using the matrix-assisted laser desorption/ionization time-of-flight analyzer (Bruker).

Contamination was defined in one of two ways: ≥1 CFU of a microorganism of gut or oral origin (MGO); or ≥20 CFU/20 mL of any microorganisms, including those of waterborne and skin origin (AM20) 11 12 13 . Once a duodenoscope tested positive for an MGO, it was quarantined and repeatedly sampled until it tested negative. If the duodenoscope still tested positive after three attempts, it was sent to the manufacturer for inspection and possible channel replacement. From November 2020, MGO-positive duodenoscopes underwent routine borescope inspections for channel damage and, if necessary, were sent to the manufacturer for repair.

Subgroup analysis distinguished primary contamination from persistent contamination. Primary contamination included cases with preceding negative culture sets or the emergence of different microorganisms. Persistent contamination involved the same microorganisms at species level across consecutive culture sets. Subgroup analysis excluded culture sets from duodenoscopes with no patient exposure between sets.

### Data collection

A sample size was not calculated as this study involved retrospectively retrieved data and was not designed to detect a predefined difference. Duodenoscope usage data were extracted from the endoscopy documentation system Endobase (Olympus) and the electronic patient records. All available culture set data of the Pentax ED34-i10T2 duodenoscopes were extracted from the electronic laboratory information system of the Department of Medical Microbiology and Infectious Diseases. The culture set result was determined by combining the five duodenoscope sample site results. Additionally, the duodenoscopes’ repair history and maintenance records were obtained from the manufacturer.

### Statistical analyses

All analyses were performed using R version 4.1.3 14 . Categorical variables are presented as absolute or relative frequencies (%), while continuous variables are expressed as the median with the first and third quartile (Q1, Q3), or as the mean (SD). Point estimates of contamination are accompanied by Wilson score confidence intervals (95%CIs).

To analyze the effect of the EPP on contamination with MGOs or by the AM20 definition, logistic mixed-effects regression models were employed, with endoscope-specific random intercepts incorporated to account for potential correlation between observations of the same duodenoscope 15 . The following covariates, were included: duodenoscope usage since the preceding culture set, preceding culture set positive for an MGO, preceding culture set positive by AM20, and duodenoscope usage since the last biopsy channel replacement. The covariates were selected based on the existing literature and clinical expertise. To facilitate model estimation, duodenoscope usage since the preceding culture was divided by 10, and duodenoscope usage since the last biopsy channel replacement was divided by 30.

A subgroup analysis was conducted to assess the impact of the EPP specifically on primary contamination. Additionally, we used mixed-model analyses to compare the odds of contamination per sample site. To adjust for the increased risk of type-I errors due to multiple testing, we applied the Bonferroni correction and set the significance threshold to *P* <0.004.

## Results

### Culture characteristics

A total of 257 culture sets were collected from eight Pentax ED34-i10T2 duodenoscopes. There were 176 culture sets (68.5%) collected pre-intervention (March, 2018 to December 15, 2020) and 81 culture sets (31.5%) collected during the intervention (December 15, 2020 to June 2022). Table 1 presents an overview of the culture characteristics. The cultured MGOs are listed in **Tables 1s** and **2s** , and the microorganisms cultured by the AM20 definition in **Tables 3s** and **4s** .

**Table:** **Table 1** Contamination of duodenoscopes before and after the introduction of the Endoss Push and Pull brush.

	Pentax single-use brush (CS5522A) (176 culture sets)				Endoss Push and Pull brush (JPP50) (81 culture sets)			
Contamination	MGO		AM20		MGO		AM20	
	No	Yes	No	Yes	No	Yes	No	Yes
Duodenoscope* culture sets (n=257), n (%) [95%CI]	96 (54.5) [47.2–61.7]	80 (45.5) [38.3–52.8]	59 (33.5) [27.0–40.8]	117 (66.5) [59.2–73.0]	67 (82.7) [73.1–89.4]	14 (17.3) [10.6–26.9]	6 (7.4) [3.4–15.2]	75 (92.6) [84.8–96.6]
Sample sites (n=1285), n (%) [95%CI]	710 (80.7) [77.9–83.2]	170 (19.3) [16.8–22.1]	628 (71.4) [68.3–74.3]	252 (28.6) [25.7–31.7]	390 (96.4) [94.0–97.7]	15 (3.6) [2.3–6.0]	218 (53.8) [49.0–58.6]	187 (46.2) [41.4–51.0]
Air/water channel (n=257), n (%) [95%CI]	169 (96.0) [92.0– 98.1]	7 (4.0) [1.9–8.0]	161 (91.5) [86.4–94.8]	15 (8.5) [5.2–13.6]	79 (97.5) [91.4–99.3]	2 (2.5) [0.7–8.6]	79 (97.5) [91.4–99.3]	2 (2.5) [0.7–8.6]
Biopsy channel (n=257), n (%) [95%CI]	125 (71.0) [63.9–77.2]	51 (29.0) [22.8–36.1]	90 (51.2) [43.8–58.4]	86 (48.8) [41.2–56.2]	78 (96.3) [89.7–98.7]	3 (3.7) [1.3–10.3]	8 (9.9) [5.1–18.3]	73 (90.1) [81.7–94.9]
Brush (n=257), n (%) [95%CI]	122 (69.3) [62.2–75.7]	54 (30.7) [24.3–37.8]	113 (64.2) [56.9–70.9]	63 (35.8) [29.1–43.1]	72 (89.9) [80.2–94.0]	9 (11.1) [6.0–19.8]	38 (46.9) [36.4–57.7]	43 (53.1) [42.3–63.6]
Forceps elevator (n=257), n (%) [95%CI]	171 (97.1) [93.5–98.8]	5 (2.9) [1.2–6.5]	169 (96.5) [92.0–98.1]	7 (3.5) [1.9–8.0]	80 (98.8) [93.3–99.9]	1 (1.2) [0.1–6.7]	79 (97.5) [91.4–99.3]	2 (2.5) [0.7–8.6]
Suction channel (n=257), n (%) [95%CI]	123 (69.9) [62.7–76.2]	53 (30.1) [23.8–37.3]	95 (54.0) [46.6–61.2]	81 (46.0) [38.8–53.4]	79 (97.5) [91.4–99.3]	2 (2.5) [0.7–8.6]	14 (17.3) [10.1–26.9]	67 (82.7) [73.1–89.4]
Number of MGOs identified per culture set, median [Q1, Q3]	–	1.0 [1.0, 2.0]	1.0 [1.0, 2.0]	1.0 [1.0, 2.0]	–	1.0 [1.0, 1.0]	1.0 [1.0, 1.0]	1.0 [1.0, 1.75]
Number of AM20 identified per culture set, median [Q1, Q3]	2.0 [1.0, 4.0]	2.0 [1.0, 3.0]	–	2.0 [1.0, 3.0]	3.0 [2.0, 4.0]	4.0 [2.75, 5.0]	–	3.0 [2.0, 5.0]
Preceding culture set positive with an MGO (n=91), n (%)	36 (45.6%)	43 (54.4%)	31 (39.2%)	48 (60.8%)	12 (85.7%)	2 (14.3%)	2 (14.3%)	12 (85.7%)
Preceding culture set positive by AM20 (n=192), n (%)	59 (50.9%)	57 (49.1%)	32 (27.6%)	84 (72.4%)	63 (82.9%)	13 (17.1%)	6 (7.9%)	70 (92.1%)
Days since last culture set, median [Q1, Q3)	22.0 [13.0, 42.0]	15.0 [9.0, 36.0]	21.0 [12.0, 39.5]	20.0 [12.0, 37.0]	29.0 [19.0, 71.0]	71.0 [25.75, 89.0]	69.0 [43.0, 85.25]	29.0 [19.0, 71.0]
Number of uses since preceding culture set, median [Q1, Q3]	3.0 [0.0, 10.0]	6.0 [0.0, 12.25]	1.0 [0.0, 8.5]	6.0 [1.0, 14.0]	7.0 [2.5, 14.0]	11.0 [3.75, 19.75]	16.5 [1.75, 29.0]	7.0 [3.0, 13.5]
Number of uses since biopsy channel replacement, median [Q1, Q3]	48.0 [19.5, 95.0]	65.0 [23.5, 91.5]	48.0 [22.0, 75.0]	56.0 [21.0, 112.0]	78.0 [12.5, 121.0]	31.5 [6.75, 151.25]	55.5 [7.25, 115.75]	76.0 [11.5, 134.0]
Days since last biopsy channel replacement, median [Q1, Q3]	185.5 [67.75, 350.0]	178.0 [85.5, 287.25]	173.0 [92.5, 274.0]	202.0 [64.0, 353.0]	279.0 [138.0, 462.0]	231.0 [77.5, 554.5]	224.5 [39.75, 350.0]	270.0 [131.0, 506.5]
AM20, any microbial growth with ≥20 colony forming units (CFUs)/20 mL, including waterborne or skin-type microorganisms; MGO, ≥1 CFU of a microorganism of gut or oral microorganism; Q1, quartile 1; Q3, quartile 3. * Pentax ED34-i10T2.								

### Contamination with MGOs

**Fig. 2:**
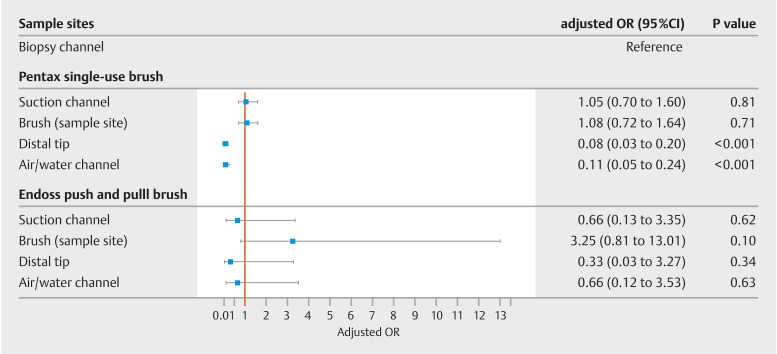
Forrest plot with results of mixed-model analysis of duodenoscope sample site contamination with MGOs by type of brush used during manual cleaning. OR, odds ratio; MGO, microorganism of gut or oral origin.

The introduction of the EPP statistically significantly reduced the odds of contamination with an MGO (adjusted odds ratio [aOR] 0.25, 95%CI 0.11–0.58; *P* =0.001) ( Fig. 1 ). We did not find a statistically significant association between the odds of contamination with an MGO and duodenoscope usage since the preceding culture set (aOR 1.10, 95%CI 0.91–1.32; *P* =0.33) or biopsy channel replacement (aOR 1.01, 95%CI 0.89–1.16; *P* =0.84). Although not statistically significant, a preceding culture set positive with an MGO seemed to increase the odds of contamination with an MGO in the subsequent culture set ( Fig. 1 ). This effect was similar in our subgroup analysis studying only primary contamination ( **Fig. 2s** ).

During the period that the Pentax single-use brush was used, the distal tip (aOR 0.08, 95%CI 0.03–0.20; *P* <0.001) and air/water channel (aOR 0.11, 95%CI 0.05–0.24; *P* <0.001) were associated with lower odds of contamination with an MGO compared with the biopsy channel ( Fig. 2 ). In the EPP period, the brush pulled through the biopsy and suction channels had higher odds of being contaminated, although this effect was not statistically significant (aOR 3.25, 95%CI 0.81–13.01; *P* =0.10).

### Contamination according to the AM20 definition

The use of the EPP increased the odds of a positive culture set by AM20 (aOR 4.43, 95%CI 1.57–12.48; *P* =0.005), but did not reach statistical significance after correction for multiple testing ( Fig. 1 ). This effect was also slightly reduced in the subgroup analysis (aOR 3.05, 95%CI 1.03–9.04; *P* =0.04) ( **Fig. 2s** ).

**Fig. 3:**
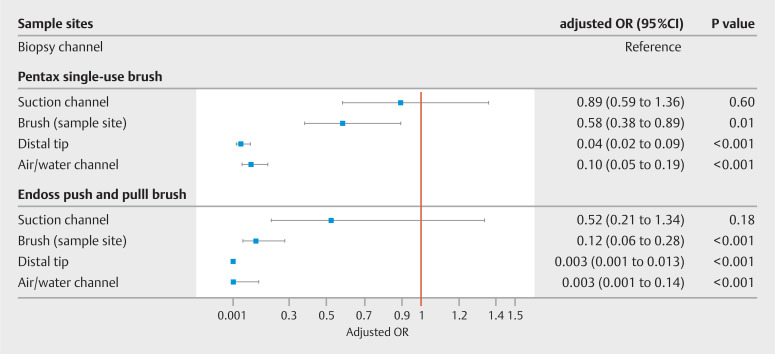
Forrest plot with results of mixed-model analysis of duodenoscope sample site contamination according to the AM20 definition by type of brush used during manual cleaning. OR, odds ratio; AM20, any microbial growth with ≥20 colony forming units/20 mL, including waterborne or skin-type microorganisms.

Duodenoscope usage was not statistically significantly associated with increased odds of contamination by AM20 ( Fig. 1 ). Although not statistically significant, a preceding culture set positive by AM20 was associated with higher odds of contamination in the subsequent culture set (aOR 1.86, 95%CI 0.94–3.69; *P* =0.08). Irrespective of the cleaning brush, the distal tip, air/water channel, and culture of the brush were associated with lower odds of contamination according to the AM20 definition compared with the biopsy channel ( Fig. 3 ).

## Discussion

After the introduction of the EPP for manual cleaning, we observed a 28.2 percentage point reduction in contamination with MGOs in Pentax ED34-i10T2 duodenoscopes. This is a remarkable finding, which has important clinical relevance. Literature reports on outbreaks highlight the risks associated with contaminated duodenoscopes. Balan et al. documented 24 outbreaks, involving 490 patients and resulting in over 30 deaths 3 . The minimum base risk of exogenous duodenoscope infections per ERCP procedure has been estimated to be 0.01% 16 . Contamination with an MGO indicates inadequate reprocessing and can occur even in the absence of identified reprocessing breaches 17 . These findings highlight the importance of innovative approaches to improve reprocessing outcomes.

In our study, *Pseudomonas aeruginosa* was the most commonly identified MGO, accounting for 14.4% of culture sets (37/257). *P. aeruginosa* is notorious for its ability to form biofilms in challenging environments, which demonstrate a certain level of tolerance to commonly used disinfectants in HLD.

Before the intervention, the duodenoscope contamination rate was 45.4%, significantly higher than the 22.5% reported in a recent meta-analysis 4 . We hypothesize that multiple duodenoscopes harbored a robust *P. aeruginosa* biofilm, contributing to the elevated contamination rate. The introduction of the EPP may have eliminated the biofilm, as only one culture set tested positive for *P. aeruginosa* after its implementation. The EPP’s design, incorporating an additional sweeper, likely improves circumferential sealing of the duodenoscope channels. This could disrupt biofilm formation and allow the disinfecting agents used during HLD to reach and eliminate the embedded bacteria.

Although not statistically significant after correcting for multiple testing, the introduction of the EPP led to an increase of culture sets contaminated by AM20, up to 90%. Even though the clinical significance of AM20 contamination is likely low, the biomatrix of environmental flora may protect MGOs during HLD 5 . The increase in AM20 contamination was observed specifically in sample sites treated with the EPP, namely the biopsy and suction channels. We suggest that the sweeper of the EPP becomes contaminated according to the AM20 definition during the manual cleaning process and subsequently contaminates the duodenoscope channels.

Duodenoscope usage or biopsy channel replacement did not seem to influence the odds of contamination with an MGO or by the AM20 definition. This is in line with the findings of Rauwers et al. 13 . Borescope studies have shown that endoscope biopsy channels are often damaged, which increases with use and has been associated with higher bacterial attachment 18 19 . However, the risk of channel damage may depend less on the frequency of use and more on ERCP characteristics, such as the instruments used.

This study has limitations associated with its before-and-after design 20 . Firstly, the order in which the brushes were used was not randomized, and no control group was available. Therefore, we cannot establish a causal relationship between the reduction in MGO contamination and the implementation of the EPP. Additionally, as this study was retrospective, important information, such as the drying time after reprocessing, the surveillance methods employed, and adherence to reprocessing and sampling protocols, was not recorded. This may have led to biased estimates of the impact of using the EPP. Furthermore, it is a single-site study and the EPP was used with only one type of duodenoscope, limiting the generalizability of our findings to other settings, types or brands of scope.

In conclusion, in this study, the introduction of the EPP was associated with significantly lower odds of contamination with an MGO in Pentax ED34-i10T2 duodenoscopes. Therefore, this seems a promising intervention to reduce contamination rates of ready-to-use duodenoscopes and improve the prevention of duodenoscope-associated infections. Future prospective multicenter studies in multiple duodenoscope brands should be performed to confirm these observations.

## Supplementary Material

Supplementary material Zusatzmaterial
