# Supplementary material for: Effect of a novel endoscope cleaning brush on duodenoscope contamination
Source: Endoscopy. 2023 Dec 5;56(3):198–204. doi: 10.1055/a-2193-4481 (PMC11583001; doi:10.1055/a-2193-4481)
Supplement: Supplementary file 1 — Supplementary material [file 10-1055-a-2193-4481_22093529.pdf_suppl.pdf.pdf]

Supplementary material

Effect of a novel endoscope cleaning brush on duodenoscope contamination

Koen van der Ploeg, Cynthia P. Haanappel, Anne F. Voor in 't holt, Woutrinus de Groot, Adriana J. C. Bulkmans, Nicole S. Erler, Bibi C.G.C. Mason-Slingerland, Margreet C. Vos, Marco J. Bruno, Juliëtte A. Severin

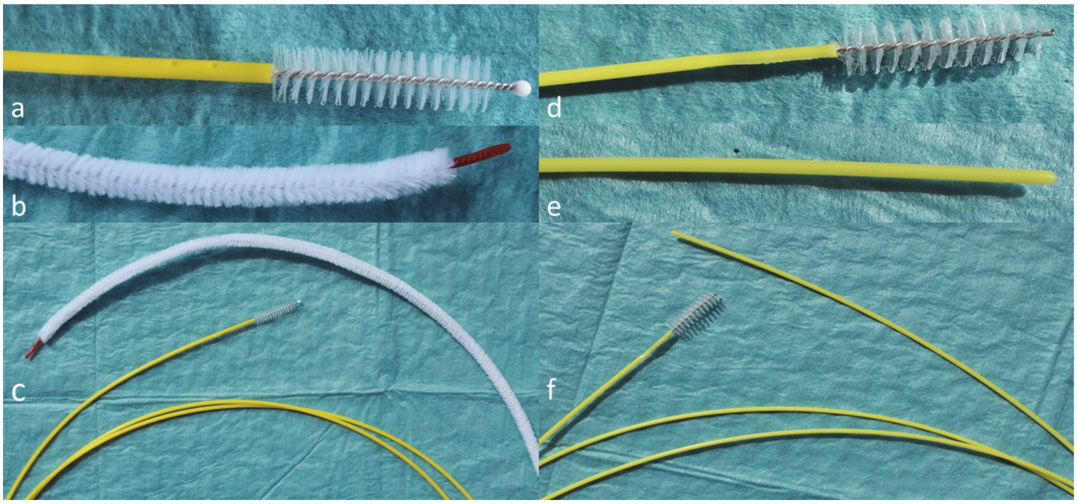

Figure 1s Images showing a comparison of: **a–c** Endoss Push and Pull brush (JPP50) and **d–f** Pentax single-use brush (CS5522A).
(a) Close-up view of the tip with brush, (b) close-up view of the distal sweeper, and (c) overall view. Technical details: Length 230cm, compatible with endoscope channels of Ø 2.1-4.5mm.
(d) Close-up view of the tip with brush, (e) close-up view of the distal tip, and (f) overall view. Technical details: Length 210cm, compatible with endoscope channels of Ø 2.0-5.0mm

Supplementary material

Table 1s Gastrointestinal microorganisms identified in culture sets using the different cleaning brushes

Gastrointestinal microorganisms independent of CFU	Total no. of culture sets (n=257)	Pentax single-use brush (n= 176)	Endoss Push and Pull brush (n= 81)
Pseudomonas aeruginosa	37 (14.4%)	36 (20.5%)	1 (1.2%)
Staphylococcus aureus	11 (4.3%)	8 (4.5%)	3 (3.7%)
Stenotrophomonas maltophilia	11 (4.3%)	11 (6.2%)	0 (0%)
Enterobacter cloacae complex	10 (3.9%)	9 (5.1%)	1 (1.2%)
Klebsiella pneumoniae	4 (1.6%)	3 (1.7%)	1 (1.2%)
Enterobacter aerogenes	2 (0.8%)	2 (1.1%)	0 (0%)
Enterococcus faecalis	2 (0.8%)	2 (1.1%)	0 (0%)
Acinetobacter pitii	1 (0.4%)	1 (0.6%)	0 (0%)
Citrobacter braakii	1 (0.4%)	1 (0.6%)	0 (0%)
Citrobacter freundii	1 (0.4%)	1 (0.6%)	0 (0%)
Enterobacter spp.	1 (0.4%)	1 (0.6%)	0 (0%)
Enterococcus faecium	1 (0.4%)	1 (0.6%)	0 (0%)
Escherichia coli	1 (0.4%)	1 (0.6%)	0 (0%)
Yeast and molds			
Candida parapsilosis	3 (1.2%)	3 (1.7%)	0 (0.0%)
Yeast NFI	1 (0.4%)	1 (0.6%)	0 (0.0%)
Aspergillus fumigatus	1 (0.4%)	1 (0.6%)	0 (0.0%)
Aspergillus niger complex	1 (0.4%)	0 (0.0%)	1 (1.2%)
Candida orthopsilosis	1 (0.4%)	0 (0.0%)	1 (1.2%)
Number of culture sets positive with gastrointestinal microorganisms	75 (29.2%)	68 (38.6%)	7 (8.6%)

Culture sets can contain multiple gastrointestinal microorganisms; therefore, the number of positive culture sets is not necessarily equal to the sum of individual microorganisms identified. CFU, Colony Forming Units; NFI, not further identified; no., Number; spp., species.

Supplementary material

Table 2s Oral microorganisms identified in culture sets using the different cleaning brushes

Oral microorganisms independent of CFU	Total no. of culture sets (n=257)	Pentax single-use brush (n= 176)	Endoss Push and Pull brush (n= 81)
Moraxella spp.	5 (1.9%)	5 (2.8%)	0 (0%)
Moraxella osloensis	4 (1.6%)	3 (1.7%)	1 (1.2%)
Neisseria spp.	4 (1.6%)	2 (0.8%)	2 (2.5%)
Gram negative cocci NFI	3 (1.2%)	1 (0.6%)	2 (2.5%)
Rothia dentocariosa	3 (1.2%)	2 (0.8%)	1 (1.2%)
Rothia mucilaginosa	3 (1.2%)	2 (0.8%)	1 (1.2%)
Actinomyces oris	2 (0.8%)	1 (0.6%)	1 (1.2%)
Neisseria subflava	2 (0.8%)	1 (0.6%)	1 (1.2%)
Gemella haemolysans	1 (0.4%)	0 (0%)	1 (1.2%)
Haemophilus parainfluenzae	1 (0.4%)	1 (0.6%)	0 (0%)
Neisseria flavescens	1 (0.4%)	1 (0.6%)	0 (0%)
Neisseria mucosa	1 (0.4%)	0 (0%)	1 (1.2%)
Rothia amarae	1 (0.4%)	1 (0.6%)	0 (0%)
Rothia spp.	1 (0.4%)	0 (0%)	1 (1.2%)
Streptococcus gordonii	1 (0.4%)	1 (0.6%)	0 (0%)
Streptococcus mitis	1 (0.4%)	1 (0.6%)	0 (0%)
Streptococcus parasanguinis	1 (0.4%)	1 (0.6%)	0 (0%)
Streptococcus spp.	1 (0.4%)	1 (0.6%)	0 (0%)
Streptococcus vestibularis	1 (0.4%)	1 (0.6%)	0 (0%)
Number of culture sets positive with oral microorganisms	26 (10.1%)	19 (10.8%)	7 (8.6%)

Culture sets can contain multiple oral microorganisms; therefore, the number of positive culture sets is not necessarily equal to the sum of individual microorganisms identified. A microorganism was considered NFI when MALDI-TOF identification was not possible. CFU, Colony Forming Units; NFI, Not further identified; no., Number; spp., species

Supplementary material

Table 3s Water type microorganisms identified in culture sets using the different cleaning brushes

Water microorganisms ≥20CFU/20mL	Total. of culture sets (n=257)	Pentax single use brush (n= 176)	Endoss Push and Pull brush (n= 81)
Achromobacter xylosoxidans	8 (3.1%)	6 (3.4%)	2 (2.5%)
Aeromicrobium spp.	2 (0.8%)	2 (1.1%)	0 (0.0%)
Agrobacterium radiobacter	12 (4.7%)	8 (4.5%)	4 (4.9%)
Agrobacterium spp.	3 (1.2%)	2 (1.1%)	1 (1.2%)
Arthrobacter spp.	1 (0.4%)	1 (0.6%)	0 (0.0%)
Brevundimonas diminuta	0 (0.0%)	0 (0.0%)	0 (0.0%)
Brevundimonas spp.	2 (0.8%)	1 (0.6%)	1 (1.2%)
Chryseobacterium spp.	26 (10.1%)	13 (7.4%)	13 (16.0%)
Cupriavidus spp.	7 (2.7%)	2 (1.1%)	5 (6.2%)
Delftia acidovorans	3 (1.2%)	3 (1.7%)	0 (0.0%)
Gram negative rods	48 (18.7%)	22 (12.5%)	26 (32.1%)
Methylobacterium spp.	70 (27.2%)	39 (22.2%)	31 (38.3%)
Microbacterium oxydans	6 (2.3%)	4 (2.3%)	2 (2.5%)
Microbacterium spp.	25 (9.7%)	8 (4.5%)	17 (21.0%)
Ochrobactrum anthropi	23 (8.9%)	22 (12.5%)	1 (1.2%)
Ochrobactrum spp.	1 (0.4%)	1 (0.6%)	0 (0.0%)
Paracoccus spp.	3 (1.2%)	1 (0.6%)	2 (2.5%)
Paracoccus yeei	36 (14.0%)	11 (6.2%)	25 (30.9%)
Pseudoarthrobacter spp.	2 (0.8%)	2 (1.1%)	0 (0.0%)
Pseudomonas alcaligenes	2 (0.8%)	2 (1.1%)	0 (0.0%)
Pseudomonas stutzeri	11 (4.3%)	10 (5.7%)	1 (1.2%)
Pseudoxanthomonas mexicana	37 (14.4%)	7 (4.0%)	30 (37.0%)
Pseudoxanthomonas spp.	4 (1.6%)	0 (0.0%)	4 (4.9%)
Sphingobacterium spiritivorum	4 (1.6%)	2 (1.1%)	2 (2.5%)
Sphingomonas koreensis	17 (6.6%)	9 (5.1%)	8 (9.9%)
Sphingomonas parapaucimobilis	1 (0.4%)	1 (0.6%)	0 (0.0%)
Sphingomonas paucimobilis	3 (1.2%)	3 (1.7%)	0 (0.0%)
Sphingomonas spp.	5 (1.9%)	3 (1.7%)	2 (2.5%)
Sphingopyxis terrae	2 (0.8%)	2 (1.1%)	0 (0.0%)

AM20, microbial growth with ≥20 CFU/20 mL of water or skin type microorganisms; CFU, colony forming units; spp., species

Supplementary material

Table 4s Skin type microorganisms identified in culture sets using the different cleaning brushes

Skin microorganisms ≥20CFU/20mL	Total. of culture sets (n=257)	Pentax single use brush (n= 176)	Endoss Push and Pull brush (n= 81)
Bacillus cereus	27 (10.5%)	18 (10.2%)	9 (11.1%)
Bacillus spp.	3 (1.2%)	1 (0.6%)	2 (2.5%)
Brevibacterium casei	22 (8.6%)	18 (10.2%)	4 (4.9%)
Brevibacterium spp.	2 (0.8%)	1 (0.6%)	1 (1.2%)
Cellulosimicrobium cellulans	9 (3.5%)	2 (1.1%)	7 (8.6%)
Gram positive cocci	7 (2.7%)	3 (1.7%)	4 (4.9%)
Gram positive rods	27 (10.5%)	14 (8.0%)	13 (16.0%)
Gram unstable rods	7 (2.7%)	3 (1.7%)	4 (4.9%)
Micrococcus luteus	6 (2.3%)	6 (3.4%)	0 (0.0%)
Staphylococcus epidermidis	7 (2.7%)	4 (2.3%)	3 (3.7%)
Staphylococcus hominis	2 (0.8%)	2 (1.1%)	0 (0.0%)
Staphylococcus lugdunensis	2 (0.8%)	1 (0.6%)	1 (1.2%)
Staphylococcus spp.	5 (1.9%)	5 (2.8%)	0 (0.0%)
Staphylococcus warneri	21 (8.2%)	9 (5.1%)	12 (14.8%)

AM20, microbial growth with ≥20 CFU/20 mL of water or skin type microorganisms; CFU, colony forming units; spp., species

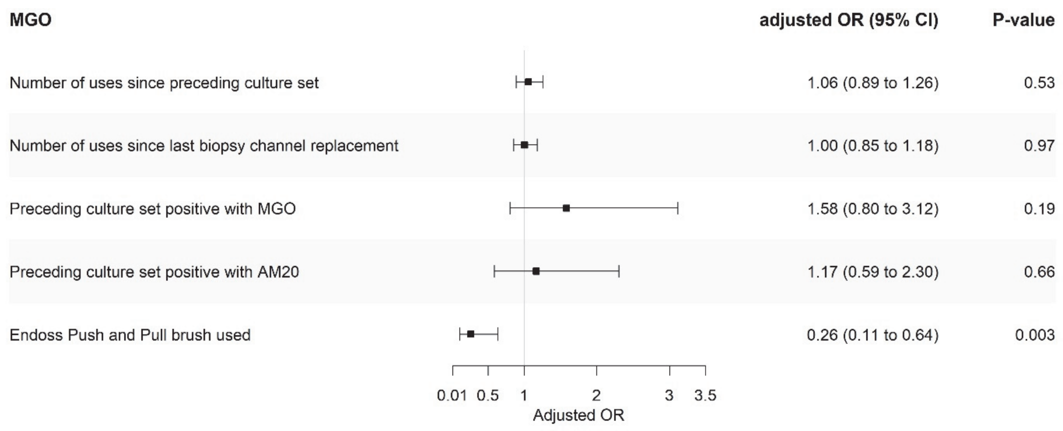

Figure 2s Subgroup analysis of infection with microorganisms of gut or oral origin (MGOs).
